# Population density gratings induced by few-cycle optical pulses in a resonant medium

**DOI:** 10.1038/s41598-017-12267-w

**Published:** 2017-09-29

**Authors:** R. M. Arkhipov, A. V. Pakhomov, M. V. Arkhipov, I. Babushkin, A. Demircan, U. Morgner, N. N. Rosanov

**Affiliations:** 1 St. Petersburg State University, 7/9 Universitetskaya nab., St. Petersburg, 199034 Russia; 2 Max Planck Institute for the Science of Light, Staudtstraße 2, 91052 Erlangen, Germany; 3 ITMO University, Kronverkskiy prospekt, 49, St. Petersburg, 197101 Russia; 4 Samara National Research University, Moskovskoye Shosse 34, Samara, 443086 Russia; 5 Department of Theoretical Physics, Lebedev Physical Institute, Novo-Sadovaya str. 221, Samara, 443011 Russia; 6 Institute of Quantum Optics, Leibniz University Hannover, Welfengarten 1, 30167 Hannover, Germany; 7 Max Born Institute, Max Born Str. 2a, Berlin, 12489 Germany; 8 Hannover Centre for Optical Technologies, Nienburger Str. 17, 30167 Hannover, Germany; 9 Vavilov State Optical Institute, Kadetskaya liniya V.O. 5/2, St. Petersburg, 199053 Russia; 10 Ioffe Physical Technical Institute, Politekhnicheskaya ul. 26, St. Petersburg, 194021 Russia

## Abstract

Creation, erasing and ultrafast control of population density gratings using few-cycle optical pulses coherently interacting with resonant medium is discussed. In contrast to the commonly used schemes, here the pulses do not need to overlap in the medium, interaction between the pulses is mediated by excitation of polarization waves. We investigate the details of the dynamics arising in such ultrashort pulse scheme and develop an analytical theory demonstrating the importance of the phase memory effects in the dynamics.

## Introduction

Population gratings can be created in any nonlinear medium using overlapping light beams^1^. In this situation, modulation of intensity due to interference leads to modification of the atomic level inversion and thus to a population density grating. Such gratings have found various applications in optics. For example, in ref.^2,3^ diffraction of radiation on electromagnetically induced population gratings was studied experimentally. In ref^4^. it was shown that population density gratings can lead to a lasing without inversion. Transient Bragg diffraction was observed in ref.^5^. Also, applications in nonlinear optics and spectroscopy are to be mentioned, such as detection of nonlinear susceptibilities^6^, quantum dots spectroscopy^7,8^ and for the molecules dynamics study^9^.

However, all the applications mentioned above require rather long pulses. Recently, significant progress in the generation of extremely short light pulses has been achieved, which allowed to approach the single optical cycle or even less^10–13^. Such pulses are used to study ultrafast processes in matter, for instance real time probing of electrons dynamics in some media^14–17^. The pulse duration *τ*_*p*_ in this case is typically much smaller than the polarization relaxation time *T*_2_, *τ*_*p*_ ≪ *T*_2_ and thus coherent light-matter interaction arises^18^.

The presence of a “phase memory” in this case changes significantly the dynamics of the pulse propagation. For instance, if the pulse amplitude is strong enough, it can propagate in a resonant absorbing medium practically without losses in the regime of self-induced transparency (SIT)^19^. Due to the presence of phase memory, a direct intersection of the pulses for grating creation^20,21^ is not required anymore. The reason for this is the appearance of polarization oscillations at the optical frequency, which remains after the pulse passage. This induced polarization grating can interact with other pulses, leading to modification of population inversion and thus to a grating. Such gratings were observed in the first experiments on photon echo on the nanosecond time scale^20^ and were used in the so-called echo holography^21^.

On the other hand, propagation of extremely short optical pulses in nonlinear media is well-studied so far, but mostly under the conditions where the central frequency of the pulse is far from resonance transition of the medium (see refs^22–24^ and references therein), whereas interaction of ultrashort pulses with resonant media attracted much less attention so far. For instance, coherent propagation and collision of few-cycle optical pulses with a duration *τ*_*p*_ ≪ *T*_2_ in a resonant medium^25–36^ were predicted theoretically but have not yet been supported by experiments. Formation and dynamics of population density gratings in the coherent few-cycle regime has attracted only little attention^37,38^. In ref.^39^ it was shown that ultrafast pulses can create population gratings. In ref.^40^ it has been shown that polarization oscillations created by an ultrashort pulse can interact with the subsequent pulses, leading to oscillations of the population inversion.

In this paper, we provide detailed theoretical analysis of creation and control of the polarization oscillations and gratings induced in a resonant medium by non-overlapping few-cycle optical pulses. Our analysis is based on the solution of the Maxwell-Bloch equations beyond the slowly-varying envelope (SVEA) and rotating wave approximation (RWA). We use analytical methods as well as numerical modeling to analyze the conditions necessary for creation of such gratings, and to investigate their dynamics and dependence on the parameters.

The article is organized as follows: First, we consider the formation of oscillations of polarization and population inversion in a small spatial volume (or for a single atom) under the action of a pulse train both numerically and analytically, using a simple theory based on *δ*-function approach for pulse shape. After that, we improve our description by considering a more elaborated theory and confirm our predictions considering an extended spatial problem and show the possibility of a spatial grating creation and the conditions and parameters needed to accomplish the gratings. Finally, extension of the gratings dynamics beyond  the two-level approximation as well as concluding remarks are presented.

## Results

### Formation of polarization and population inversion oscillations in a single atom

In this subsection we consider analytically the interaction of a few-cycle pulse train with a single atom using a simple analytical approach introduced in^40^, by considering a pulse as a *δ*-function. This theory, although leads sometimes to unphysical results, still allows an easy understanding of the underlying dynamics. We demonstrate, how persistent oscillations of the density matrix created by a short pulse interact with the subsequent pulses in a pulse train, assuming that none of the pulses overlaps with the others.

Interaction of few-cycle optical pulses with a resonant medium can be described using the system of equations for the the density matrix of a two-level system. Neglecting relaxation terms, the corresponding system can be written in the form:

1 $$\frac{d{\rho }_{12}(t)}{dt}=i{\omega }_{0}{\rho }_{12}(t)-\frac{i{d}_{12}}{\hslash }n(t)E(t),$$

2 $$\frac{d}{dt}n(t)=\frac{4{d}_{12}E(t)}{\hslash }{\rm{Im}}({\rho }_{12}(t\mathrm{)).}$$

The equations (1 and 2) describe the dynamics of non-diagonal element *ρ*_12_(*z*, *t*) of the density matrix *ρ* as well as the population difference *n*(*t*) = *ρ*_11_(*t*)−*ρ*_22_(*t*) between the ground and excited levels. Here, *E*(*t*) is the driving field, *d*_12_ the transition dipole moment of the atoms, *ω*_0_ the resonant frequency (*λ*_0_ = 2*πc*/*ω*_0_ the corresponding wave length), *ħ* the Plank constant, and *c* the speed of light. We remark that this system is formulated without the commonly used SVEA and RWA and thus can be used to describe the pulses of any duration shorter than the relaxation times. Equations (1 and 2) were initially derived and applied for resonant two-level atomic systems^18^ but have proved their applicability for much more complicated level or band structure, in particular for various semiconductor systems^41–46^. Equations (1 and 2) can be written in the integral form as

3 $$\delta {\rho }_{12}(t)=-\frac{i{d}_{12}}{\hslash }{e}^{i{\omega }_{0}t}{\int }_{t-{t}_{-}}^{t}n(\tau ^{\prime} )E(\tau ^{\prime} ){e}^{-i\omega \tau ^{\prime} }d\tau ^{\prime} ,$$

4 $$\delta n(t)=4\frac{{d}_{12}}{\hslash }{\int }_{t-{t}_{-}}^{t}{\rm{Im}}{\rho }_{12}(\tau ^{\prime} )E(\tau ^{\prime} )d\tau ^{\prime} ,$$

where $\delta n=n(t)-n(t-{t}_{-})$, $\delta {\rho }_{12}={\rho }_{12}(t)-{\rho }_{12}(t-{t}_{-})$ is the modification of *n*, *ρ*_12_ gained by the atom between the instants of time *t* − *t*_−_ (which we assume to be before the pulse begins) and *t* (which we take after the end of the pulse).

To get an analytical insight into the dynamics described by Eqs (3 and 4) we assume now the driving field *E*(*t*) in the form of a *δ*-function which would correspond to a pulse with the “infinitely small” width. That is, we define:

5 $${E}_{i}(t)=\frac{\hslash {\theta }_{i}}{{d}_{12}}\delta (t-{\tau }_{i}),$$

for the *i*-th pulse. Here *τ*_*i*_ is the delay of the *i*-th pulse with respect to the first one and *θ*_i_ is related to the amplitude of the pulse and has the meaning of the pulse area. In contrast, in the framework of SVEA and RWA the pulse area is defined^18,19^, as

6 $${\rm{\Phi }}(t,z)=\frac{{d}_{12}}{\hslash }{\int }_{-{\rm{\infty }}}^{t}{\mathscr{E}}({t}^{^{\prime} },z)d{t}^{^{\prime} },$$

where *ε*(*t*, *z*) is the pulse envelope. In particular, a pulse with the area *π*/2 fully saturates the medium, i.e. equalizes the levels’ populations (the medium is assumed to be in the ground state before the pulse), and a pulse with the area *π* fully inverts it. For very short pulses, in contrast, a real-valued envelope is not well defined.

Taking the first pulse in the form ${E}_{1}(t)=(\hslash {\theta }_{1}/{d}_{12})\delta (t)$ and using the equations (3 and 4), we obtain for the population inversion ${n}_{1}(t)\equiv {n(t)|}_{t > 0}$ and the non-diagonal density matrix element ${\rho }_{1}(t)\equiv {{\rho }_{12}(t)|}_{t\mathrm{ > 0}}$ after the first pulse:

7 $${\rho }_{1}(t)=-i{\theta }_{1}{e}^{i{\omega }_{0}t},$$

8 $${n}_{1}(t)=-4{\theta }_{1}^{2}+1.$$

It is worth noting that Eqs (7 and 8) were derived in the assumption that the modification of the polarization and population inversion of the medium ar﻿e independent of each oth﻿er during the action of the pulse. This implies that Eqs (7 and 8) can be expected to yield quantitatively correct results only for small *θ*_1_ . For the pulses with *θ*_1_ ∼ 1 this simplified theory can exhibit unphysical results as it may be seen from Eq. (8). However, the presented approach is relatively simple and we show that it allows to qualitatively describe the most prominent features of the considered phenomena. The rigorous theory is more cumbersome and will be presented in the following subsection.

We assume that the first pulse acts as a pulse with the area *π*/2 thus leaving the medium in the state with zero inversion. From Eq. (8) the area of this pulse is *θ*_1_ = 1/2. Below we consider only pulses with θ = 1/2.

Taking the second pulse in the form: ${E}_{2}(t)=({d}_{12}\mathrm{/2}\hslash )\delta (t-\tau )$ and substituting the polarization from (7) in (4) one can obtain for the inversion after the second pulse ${n}_{2}(t)\equiv {n(t)|}_{t > \tau }$

9 $${n}_{2}(t)=-\cos ({\omega }_{0}\tau ).$$

Finally, substituting this equation into Eq. (3) it is easy to obtain the equation for the nondiagonal element of the density matrix after the second pulse ${\rho }_{2}(t)\equiv {{\rho }_{12}(t)|}_{t > \tau }$:

10 $${\rho }_{2}(t)=\frac{1}{2}\,\sin ({\omega }_{0}\tau ){e}^{i{\omega }_{0}(t-\tau )}\mathrm{.}$$

From Eq. (9) we can immediately see that the inversion depends periodically on the delay between two pulses *τ*.

**Figure 1:**
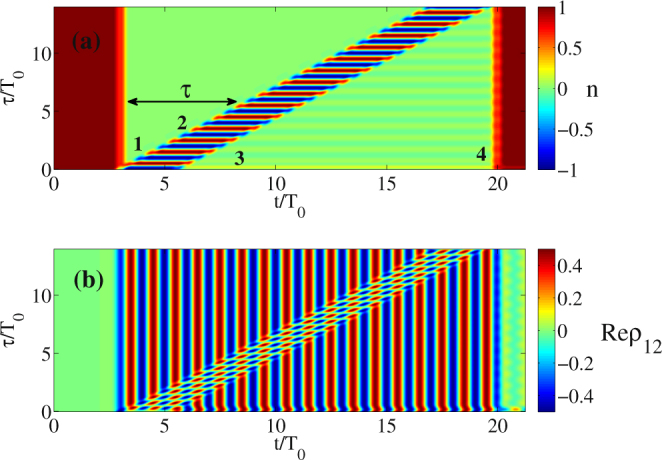
Dependence of population difference *n* (**a**) and real part of the non-diagonal element Re(*ρ*_12_) (polarization of a single atom) (**b**) on the distance *τ* between the pulses (1) and (2) and time *t*. We assume that the pulse (1) crosses the thin-layer medium at *t* = 3*T*_0_, the pulse (3) delayed to the pulse (2) by 2.5*T*_0_, and the pulse (4) delayed to the pulse (1) by 17*T*_0_. All the time durations are normalized to ${T}_{0}\,=\,2\pi /{\omega }_{0}$. The first vertical “front“ shows the pulse (1) whereas the pulses (2) and (3) form “oblique“ fronts (because the delay *τ* changes). The pulse (4) (last vertical “front“) returns the system back to the initial state. Parameters: *d*_12_ = 5 · 10^−18^ ESU, *E*_0_ = 2.6 · 10^5^ ESU, *λ*_0_ = 0.7 · 10^−4^ cm, *ω*_0_ = 2.693 · 10^15^ rad/s, *τ*_*p*_ = 7.4 · 10^−16^ s, *T*_1_ = *T*_2_ = ∞.

To confirm the results of our analytical approach we perform numerical simulations of the model equations (1 and 2) using pulses with finite durations. The electric field was taken in the form:

11 $$\begin{array}{c}E(t)={E}_{0}\exp (-{t}^{2}/{\tau }_{p}^{2})\sin \,{\omega }_{0}t\\ \quad \quad \,\,\,\,\,+{E}_{0}\exp (-{(t-\tau )}^{2}/{\tau }_{p}^{2})\sin \,{\omega }_{0}(t-\tau )\\ \quad \quad \,\,\,\,\,+{E}_{0}\exp (-{(t-{\rm{\Delta }}{\tau }_{\mathrm{2,3}}-\tau )}^{2}/{\tau }_{p}^{2})\sin \,{\omega }_{0}(t-{\rm{\Delta }}{\tau }_{\mathrm{2,3}}-\tau )\\ \quad \quad \,\,\,\,\,+{E}_{0}\exp (-{(t-{\rm{\Delta }}{\tau }_{\mathrm{1,4}})}^{2}/{\tau }_{p}^{2})\sin \,{\omega }_{0}(t-{\rm{\Delta }}{\tau }_{\mathrm{1,4}})\mathrm{.}\end{array}$$

where *τ* is the delay between the pulses 1 and 2 (see Fig. 1), whereas Δ*τ*_2,3_ and Δ*τ*_1,4_ are delays between the second and third and the first and fourth pulses, respectively, which in the present simulations were fixed to 2.5*T*_0_ and 17*T*_0_. For simplicity we assumed the pulse amplitudes *E*_0_ to be equal with E_0_ = 2.6 · 10﻿^5^﻿ ESU﻿. Figure 1 illustrates the dependence of the population inversion *n* and of the real part of *ρ*_12_ after the passage of the pulses as a function of time *t* and of the delay *τ*. It is seen from Fig. 1a that the inversion depends periodically on *τ*, which is in a good agreement with Eq. (9). Polarization oscillates with the frequency *ω*_0_ after the first pulse, see Fig. 1b. On the other hand, population inversion remains constant in both *t* and *τ* after the first pulse whereas after the second pulse remains constant only in *t*, and oscillating periodically in *τ*, which is also in agreement with Eqs (9 and 10).

As a next step, we demonstrate a possibility to erase the oscillations of *n* by launching the third pulse, entering the medium with the delay Δ*τ*_2,3_ with respect to the second one. Taking the expression for the third pulse in the form ${E}_{3}(t)=(\hslash \mathrm{/2}{d}_{12})\delta (t-\tau -{\rm{\Delta }}{\tau }_{\mathrm{2,3}})$ we obtain for the nondiagonal element of density matrix:

12 $${\rho }_{3}(t)=\mathrm{1/2}{e}^{i{\omega }_{0}(t-\tau )}(i{e}^{-i{\omega }_{0}{\rm{\Delta }}{\tau }_{\mathrm{2,3}}}\,\cos \,{\omega }_{0}\tau +\,\sin \,{\omega }_{0}\tau )\mathrm{.}$$

We note that

13 $${\rho }_{3}(t)=-\frac{i}{2}{e}^{i{\omega }_{0}t},$$

assuming ${\omega }_{0}{\rm{\Delta }}{\tau }_{\mathrm{2,3}}=\pi \mathrm{(2}m+\mathrm{1)}$, *m* = 0, 1, 2, 3…. Substituting Eq. (13) in Eq. (4) we find that inversion after the third pulse *n*_3_ has the form:

14 $${n}_{3}(t)=-\cos \,{\omega }_{0}(\tau +{\rm{\Delta }}{\tau }_{\mathrm{2,3}})-\,\cos \,{\omega }_{0}\tau =0.$$

**Figure 2:**
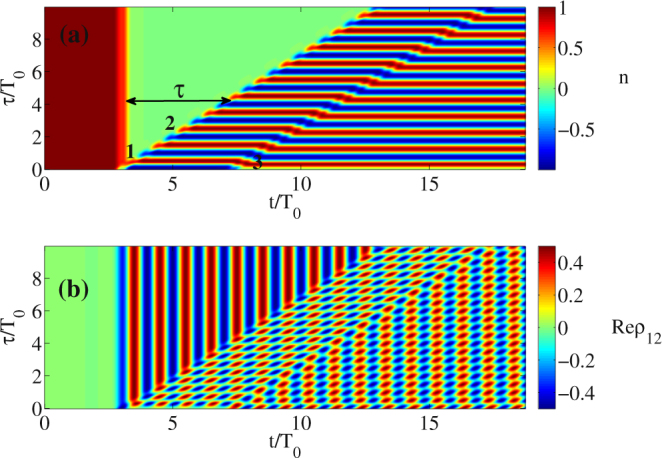
Dependence of population difference *n* (**a**) and real part of the non-diagonal element Re(*ρ*_12_) (polarization of a single atom) o﻿﻿n ti﻿me﻿ *t* and delay between the pulses *τ* (1﻿) and (2), similar to Fig. 1 but with the delay to the pulse (3) Δ*τ*_2,3_ = 4.3*T*_0_. In the this case, the pulse (3) does not erase the action of the pulses (1), (2). The other parameters are the same as in Fig. 1.

From Eq. (14) one can immediately see that the third pulse launched to the medium with an appropriate delay can erase the inversion oscillations created by the second pulse. As one can see from the numerical modeling in Fig. 1, this prediction is also held. To illsutrate the importance of the delay between the pulses, in Fig. 2 we have ta﻿ken﻿ a larger one: ${\rm{\Delta }}{\tau }_{\mathrm{2,3}}=4.3{T}_{0}$. In this case, the oscillations after the third pulse do not disappear﻿, which is in agreement with Eq. (14). Thus, we see that the delay between the pulses strongly influences the grating dynamics.

**Figure 3:**
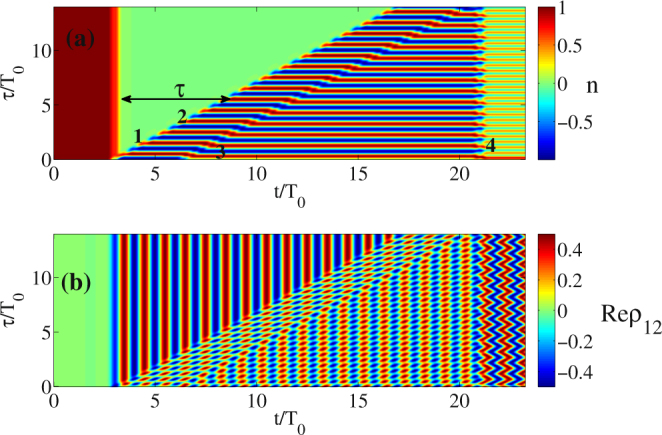
Dependence of population difference *n* (**a**) and real part of the non-diagonal element Re(*ρ*_12_) (polarization of a single atom) o﻿n ti﻿me﻿ t and delay ﻿τ between the pulses (1﻿) and (2), similar to Fig. 1 but with the delays Δ*τ*_2,3_ = 3.3*T*_0_ and Δ*τ*_1,4_ = 18*T*_0_. The parameters are the same as in Fig. 1.

Finally, we demonstrate that a fourth pulse can return the system to the initial (ground) state. Taking the expression for the pulse in the form ${E}_{4}(t)=(\hslash \mathrm{/2}{d}_{12})\delta (t-{\rm{\Delta }}{\tau }_{\mathrm{1,4}})$ (Δ*τ*_1,4_ is delay between the first and the fourth pulse), substituting the expression Eq. (13) into Eq. (4) we obtain for the inversion:

15 $${n}_{4}(t)=-\cos \,{\omega }_{0}{\rm{\Delta }}{\tau }_{\mathrm{1,4}}=\mathrm{1,}$$

for ${\omega }_{0}{\rm{\Delta }}{\tau }_{\mathrm{1,4}}=\pi \mathrm{(2}m+\mathrm{1)}$, *m* = 0, 1, 2, 3…. That is, the fourth pulse launched to the medium with the appropriate delay can bring the system back to the ground state (the state before the pulse train). This is also reproduced in Fig. 1. In general, as Eq. (14) and Eq. (15) show, oscillations remain after the action of pulses 3 and 4 if the phase is taken incorrect. This situation is visualized in Fig. 3 for Δ*τ*_1,4_ = 18*T*_0_ and Δ*τ*_2,3_ = 3.3*T*_0_.

**Figure 4:**
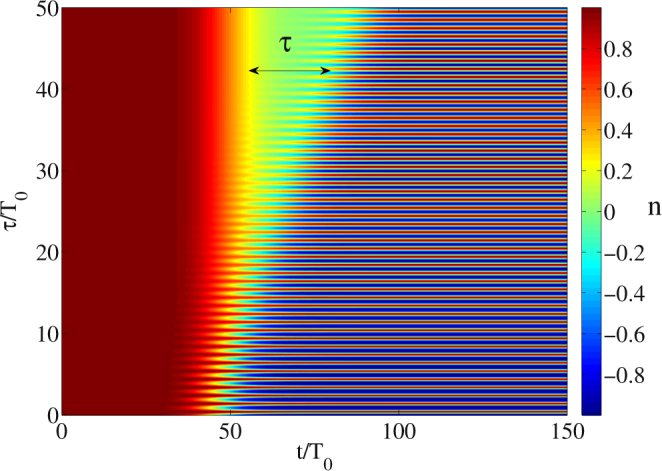
Dependence of population difference *n* on the distance *τ* between the pulses (1) and (2) and time *t* as in Fig. 1a with the similar parameters, only without the pulses (3) and (4) and for the longer pulse durations *τ*_*p*_ = 35 fs; ${E}_{0}=5.3\cdot {10}^{3}$ ESU.

Up to now we considered the switching pulses to be very short. In fact, the dynamics is also essentially the same for the pulses of arbitrary length. For instance, in Fig. 4 the population difference dynamics is shown for the case of a longer pulse of around 15 cycles (*τ*_*p*_ = 35 fs), where one can observe the same grating structure as in Fig. 1a.

To conclude this subsection, we remark that after the system is returned to the initial state, we can repeat the process from the beginning by a new set of pulses. The tunable “delayed” action  of the pulses in the train, as we can see here from the simple theory presented above, can be traced to the fact that the phase of the polarization oscillations created by the pulse depends on time, and thus, if we vary the delay between pulses, we can tune the delayed interaction between the light and polarization waves. Thus, the phase memory plays here the critical role.

### Detailed theory

The theory developed in the previous subsection allows to understand the pulse-to-pulse interaction mechanism very easily. However, it is mathematically inconsistent and valid only in the limit of pulse areas much less than unit while for greater pulse areas it gives not-unique results. In this subsection we develop a more consistent theory. We start again from Eqs (1 and 2), from which it follows that:

16 $$|{\rho }_{12}(t{)|}^{2}+\frac{n{(t)}^{2}}{4}={\rm{const}}\mathrm{.}$$

Based on Eq. (16) and denoting the constant on the right side of Eq. (16) as *A* ^2^, it is convenient to introduce real variables Φ and *φ*:

17 $$n(t)=2A\,\cos \,{\rm{\Phi }}(t),$$

18 $${\rho }_{12}(t)=A\,\sin \,{\rm{\Phi }}(t){e}^{i\phi (t)}\mathrm{.}$$

With these new variables Eqs (1–2) turn into:

19 $$\frac{d{\rm{\Phi }}(t)}{dt}=-2{\rm{\Omega }}(t)\sin \,\phi (t),$$

20 $$\frac{d\phi (t)}{dt}={\omega }_{0}-2{\rm{\Omega }}(t)\cos \,\phi (t){\rm{c}}{\rm{t}}{\rm{g}}\,{\rm{\Phi }}(t),$$

where

21 $${\rm{\Omega }}(t)=\frac{{d}_{12}E(t)}{\hslash }$$

is the Rabi oscillation frequency. Since Eqs (19–20) can not be treated analytically in general form, we will consider two limiting cases, namely “very long” and “very short” pulses.

#### A single long pulse

As a first limiting case, we consider long pulses, i.e., we assume the optical transition frequency ${\omega }_{0}$ to be much greater than the Rabi oscillation frequency ${\rm{\Omega }}(t)$:

22 $${\omega }_{0}\gg {\rm{\Omega }}(t\mathrm{).}$$

In this way we reintroduce the well-known result (see, for instance,^40^) for the sake of completeness, si﻿nc﻿e﻿ we need it later to consider a pulse train. Condition (22) means also that the excitation pulse is much longer than the single-cycle duration, what is the most common situation and does not allow to neglect the second term in Eq. (20). Indeed, since the phase *φ*(*t*) is a 2*π*-periodic function, the second term can be omitted only if its contribution to the overall phase change is much smaller than *π*. As the latter contribution turns to be proportional to the excitation pulse area according to the Eq. (20), the pulse area has to be necessary much less than *π*. We remind that if this latter condition is fulfilled (that is, we have a weak pulse), we can represent the field *E*(*t*) as a product of a slowly varying envelope *ε*(*t*) and a carrier wave sin (*ω*_0_ *t*) and obtain

23 $${\rm{\Phi }}(t)=-{\int }_{-\infty }^{t}\tilde{{\rm{\Omega }}}(t^{\prime} )dt^{\prime} ,$$

where

24 $$\mathop{{\rm{\Omega }}}\limits^{ \sim }(t)=\frac{{d}_{12}{\mathscr{E}}(t)}{\hslash }.$$

In this way we reintroduce the well-known result for long pulses in RWA approximation, so the Eq. (23) coincides with Eq. (6).

If the pulse area becomes of the order of *π*, but the pulse is still considered to be much longer than the optical period, we can expand the phase *φ*(*t*) as:

25 $$\phi (t)={\omega }_{0}(t-{\tau }_{1}){\rm{\Theta }}[t-{\tau }_{1}]+\phi ^{\prime} (t),$$

where *τ*_1_ is the time moment of the first pulse arrival, ${\rm{\Theta }}(t)$ is the Heaviside step function and *φ*′(*t*) is assumed to be a slowly varying function:

26 $$|\dot{\phi }^{\prime} (t)|\ll {\omega }_{0}\mathrm{.}$$

It is convenient to introduce the integral phase shift in Eq. (25) as follows:

27 $$\tilde{\phi }(t)=\phi ^{\prime} (t)-{\omega }_{0}{\tau }_{1}\mathrm{.}$$

We represent the electric field in Eq. (19) as:

28 $${E}_{i}(t)={\mathscr{E}}(t-{\tau }_{i})\sin ({\omega }_{0}(t-{\tau }_{i})+{\varphi }_{i}),$$

i.e., we express the electric ﻿field as a product of a slowly varying envelope *ε*(*t*) and a carrier wave where ${\varphi }_{i}$ denotes the carrier-envelope phase (CEP) of *i*-th pulse and the time moment *τ*_*i*_ of the *i*-th pulse arrival accounts for the pulse propagation effects.

Averaging Eqs (19 and 20) over the optical period and thus omitting the fast oscillating terms on right side, we obtain:

29 $$\frac{d{\rm{\Phi }}(t)}{dt}=-\tilde{{\rm{\Omega }}}(t)\cos (\tilde{\phi }(t)+{\omega }_{0}{\tau }_{i}-{\varphi }_{i}),$$

30 $$\frac{d\mathop{\phi }\limits^{ \sim }(t)}{dt}=\mathop{{\rm{\Omega }}}\limits^{ \sim }(t){\rm{c}}{\rm{t}}{\rm{g}}[{\rm{\Phi }}(t)]\sin [\mathop{\phi }\limits^{ \sim }(t)+{\omega }_{0}{\tau }_{i}-{\varphi }_{i}].$$

From Eqs (29–30) it follows, that during the action of the *i*-th pulse:

31 $$\sin \,{\rm{\Phi }}(t)\sin (\mathop{\phi }\limits^{ \sim }(t)+{\omega }_{0}{\tau }_{i}-{\varphi }_{i})={\rm{c}}{\rm{o}}{\rm{n}}{\rm{s}}{\rm{t}}.$$

Denoting the latter constant as *K*_*i*_ and expressing $\tilde{\phi }(t)$ from Eq. (31), Eq. (29) yields:

32 $$\frac{d{\rm{\Phi }}(t)}{dt}=\mp \tilde{{\rm{\Omega }}}(t)\frac{\sqrt{{\sin }^{2}{\rm{\Phi }}(t)-{K}_{i}^{2}}}{\sin \,{\rm{\Phi }}(t)}\mathrm{.}$$

Upon the integration of Eq. (32) over the whole excitation pulse duration, we get for the resulting value Φ:

33 $$\cos \,{\rm{\Phi }}=\sqrt{1-{K}_{i}^{2}}\,\sin \,(\pm \mathop{{\rm{\Theta }}}\limits^{ \sim }+\arcsin (\frac{{\cos {\rm{\Phi }}}_{0}}{\sqrt{1-{K}_{i}^{2}}})),$$

where

34 $$\tilde{{\rm{\Theta }}}={\int }_{-\infty }^{+\infty }\tilde{{\rm{\Omega }}}(t^{\prime} )dt^{\prime} $$

is the pulse area. Eq. (33) relates the population inversion distribution before and after the action of the pulse. The effective influence of the pulse is determined by the well-known expression for the pulse area^18^ (34) while the phase of the pulse is accounted for by the variable *K*_*i*_. In between the pulses according to Eqs (29 and 30) ${\rm{\Phi }}(t)={\rm{const}}$, $\tilde{\phi }(t)={\rm{const}}$.

#### A single ultrashort pulse

If the two-level medium is excited by a few-cycle pulse, two terms on the right side of the Eq. (20) can become comparable (${\rm{\Omega }}(t)\sim {\omega }_{0}$). In this case we need high pulse intensity, that is, we assume

35 $${\rm{\Omega }}(t)\gg {\omega }_{0}.$$

In this case the first term in Eq. (20) can be neglected. Although the assumption Eq. (35) is difficult for an experimental realization, it allows to get a﻿n analytical insight into the role of the Rabi frequency term on the right side of the Eq. (20), arising from the few-cycle and even subcycle pulse duration, and thus unveil the differences to the conventional SIT effects. Eqs (19 and 20) now take the form:

36 $$\frac{d{\rm{\Phi }}(t)}{dt}=-2{\rm{\Omega }}(t)\sin \,\phi (t),$$

37 $$\frac{d\phi (t)}{dt}=-2{\rm{\Omega }}(t)\cos \,\phi (t)\mathrm{ctg}\,{\rm{\Phi }}(t\mathrm{).}$$

It can be easily obtained from Eqs (36 and 37), that during the action of the pulse

38 $$\sin \,{\rm{\Phi }}(t)\cos \,\phi (t)={\rm{const}}\mathrm{.}$$

Denoting the latter constant as *B*_*i*_ and expressing *φ*(*t*) from Eq. (38), Eq. (36) yields:

39 $$\frac{d{\rm{\Phi }}(t)}{dt}=\mp 2{\rm{\Omega }}(t)\frac{\sqrt{{\sin }^{2}{\rm{\Phi }}(t)-{B}_{i}^{2}}}{\sin \,{\rm{\Phi }}(t)}\mathrm{.}$$

Upon the integration of Eq. (39) over the whole pulse duration, we get the resulting value Φ:

40 $$\cos \,{\rm{\Phi }}=\sqrt{1-{B}_{i}^{2}}\,\sin (\pm 2{\rm{\Theta }}+\arcsin (\frac{{\cos {\rm{\Phi }}}_{0}}{\sqrt{1-{B}_{i}^{2}}})),$$

where

41 $${\rm{\Theta }}={\int }_{-\infty }^{+\infty }{\rm{\Omega }}(t^{\prime} )dt^{\prime} $$

is the whole pulse area, taken now for the electric field *E*(*t*) in contrast to the pulse envelope in Eqs (23), (34). In between the pulses, according to Eqs (19 and 20) ${\rm{\Phi }}(t)={\rm{const}}$, $\delta \phi (t)={\omega }_{0}t$.

The difference between the definition of the pulse area in Eqs (34) and (41) is due to the failure of the concepts of pulse envelope and pulse area for subcycle pulses^29^. Specifically, in the approximation Eq. (35), according to Eq. (41), in order to change the state of the medium, the pulse has to contain a constant component, i.e. to be unipolar, because the Bloch vector here is “field-driven” and its free rotation is completely neglected. In contrast to the common belief, ultrashort unipolar pulses can be indeed obtained in various ways, see refs^47–52^ and reviews^53,54^. However, we remind that we use the approximation Eq. (35) to show analytically that our method works for all possible pulse durations, at least for two-level approximation. In practice, we do not need necessarily unipolar pulses.

#### A train of long pulses

Let us consider the dynamics under excitation by a *train* of non-overlapping pulses. First we conside the case of long pulses, Eq. (22). We suppose the resonant medium to be initially uninverted, what means for Eqs (17 and 18) $A=\frac{1}{2},{{\rm{\Phi }}}_{0}=0$. We consider now a strong exciting pulse, which has the pulse area of $\tilde{{\rm{\Theta }}}\sim \pi$ and can thus significantly transform the population of the medium. We then get, according to Eq. (33), for the state of the medium after the $(n+\mathrm{1)}$-th pulse:

42 $$\begin{array}{rcl}{K}_{n+1} & = & {\sin {\rm{\Phi }}}_{n}\,\sin ({\tilde{\phi }}_{n}(t)+{\omega }_{0}{\tau }_{n+1}-{\varphi }_{n+1}),\\ {\cos {\rm{\Phi }}}_{n+1} & = & \sqrt{1-{K}_{n+1}^{2}}\,\sin (\pm \tilde{{\rm{\Theta }}}+\arcsin (\frac{{\cos {\rm{\Phi }}}_{n}}{\sqrt{1-{K}_{n+1}^{2}}})),\\ \sin ({\tilde{\phi }}_{n}(t)+{\omega }_{0}{\tau }_{n+1}-{\varphi }_{n+1}) & = & \frac{{K}_{n+1}}{{\sin {\rm{\Phi }}}_{n+1}}\mathrm{.}\end{array}$$

Let us furthermore assume that all the exciting pulses have the same pulse area equal ${\tilde{{\rm{\Theta }}}}_{i}=\frac{\pi }{2}$. We take for definiteness ${\tilde{\phi }}_{0}=0$ and suppose the resonant medium to be initially uninverted, what means for Eqs (17)–(18) $A=\frac{1}{2},{{\rm{\Phi }}}_{0}=0$. After the action of the first pulse we get:

43 $$\begin{array}{ccc}\quad \quad \quad \quad \quad \,\,\,\,\,\,{K}_{1} & = & 0,\\ \quad \quad \quad \quad \,\,\,\,\cos \,\,{{\rm{\Phi }}}_{1} & = & \sin (\frac{\pi }{2}+\arcsin 1)=\,\sin \,\pi =0,\\ \sin ({\mathop{\phi }\limits^{ \sim }}_{1}+{\omega }_{0}{\tau }_{1}-{\varphi }_{1}) & = & \frac{{K}_{1}}{{\sin {\rm{\Phi }}}_{1}}=0,\end{array}$$

so the first pulse fully saturates the medium.

Let us now consider a spatially extended medium with only one spatial dimension along the pulse propagation with the propagation coordinate *z* and the phase velocity of light *c*. We assume in this subsection a medium which is unbounded in both directions. If the first pulse starts at *t* = 0 at *z* = 0, it will reach the atoms located at the spatial position *z* at the time moment:

44 $${\tau }_{1}=\frac{z}{c},$$

so the resulting phase of the polarization wave is given as:

45 $${\tilde{\phi }}_{1}=-{\omega }_{0}{\tau }_{1}+{\varphi }_{1}=-\frac{{\omega }_{0}z}{c}+{\varphi }_{1}\mathrm{.}$$

This phase distribution corresponds to a traveling wave of polarization with the wavenumber:

$${k}_{0}=\frac{{\omega }_{0}}{c},$$

which coincides with the wavenumber of the pump pulse.

For the second pulse coming at the moment *τ*_2_ with time delay Δ*τ*_1,2_ after the first one, we obtain:

46 $$\begin{array}{ccc}\quad \quad \quad \quad \quad \,\,\,\,{K}_{2} & = & {\sin {\rm{\Phi }}}_{1}\,\sin ({\mathop{\phi }\limits^{ \sim }}_{1}+{\omega }_{0}{\tau }_{2}-{\varphi }_{2})=\,\sin ({\omega }_{0}{\rm{\Delta }}{\tau }_{1,2}+{\varphi }_{1}-{\varphi }_{2}),\\ \quad \quad \quad \quad \,\,\,{\cos {\rm{\Phi }}}_{2} & = & \sqrt{1-{K}_{2}^{2}}\,\sin (\frac{\pi }{2})=\,\cos ({\omega }_{0}{\rm{\Delta }}{\tau }_{1,2}+{\varphi }_{1}-{\varphi }_{2}),\\ \sin ({\mathop{\phi }\limits^{ \sim }}_{2}+{\omega }_{0}{\tau }_{2}-{\varphi }_{2}) & = & \frac{{K}_{2}}{\sin ({\omega }_{0}{\rm{\Delta }}{\tau }_{1,2}+{\varphi }_{1}-{\varphi }_{2})}=1.\end{array}$$

In the spatially extended medium we now assume that the second pulse propagates in the opposite direction to the first one, with *L*_1,2_ being the spatial separation between the pulses at *t* = 0:

47 $${\tau }_{2}=\frac{{L}_{\mathrm{1,2}}-z}{c}\mathrm{.}$$

Then the temporal separation between the pulses at the point *z* is:

48 $${\rm{\Delta }}{\tau }_{\mathrm{1,2}}=\frac{{L}_{\mathrm{1,2}}-2z}{c}\mathrm{.}$$

Given that, Eq. (46) means that the second pulse induces a periodic grating of population inversion together with a standing wave of polarization with the phase distribution given by:

49 $${\tilde{\phi }}_{2}=\frac{\pi }{2}-{\omega }_{0}{\tau }_{2}+{\varphi }_{2}=\frac{\pi }{2}+{\varphi }_{2}+\frac{{\omega }_{0}}{c}(z-{L}_{\mathrm{1,2}}\mathrm{).}$$

For the third pulse coming at the moment *τ*_3_ with time delay Δ*τ*_2,3_ after the second one, for the density matrix at the arbitrary position *z* we obtain:

50 $$\begin{array}{c}\quad \quad \quad \quad \quad \quad \quad \,\,\,\,\,{K}_{3}={\sin {\rm{\Phi }}}_{2}\cdot \sin ({\mathop{\phi }\limits^{ \sim }}_{2}+{\omega }_{0}{\tau }_{3}-{\varphi }_{3})=\sin ({\omega }_{0}{\rm{\Delta }}{\tau }_{1,2}+{\varphi }_{1}-{\varphi }_{2})\cdot \\ \sin (\frac{\pi }{2}+{\omega }_{0}{\rm{\Delta }}{\tau }_{2,3}+{\varphi }_{2}-{\varphi }_{3})=\sin ({\omega }_{0}{\rm{\Delta }}{\tau }_{1,2}+{\varphi }_{1}-{\varphi }_{2})\cdot \cos ({\omega }_{0}{\rm{\Delta }}{\tau }_{2,3}+{\varphi }_{2}-{\varphi }_{3}),\\ \quad \quad \quad \quad \quad \quad \,\,\cos \,{{\rm{\Phi }}}_{3}=\sqrt{1-{K}_{3}^{2}}\,\sin (\frac{\pi }{2}+\arcsin (\frac{{\cos {\rm{\Phi }}}_{2}}{\sqrt{1-{K}_{3}^{2}}}))=\,\sqrt{1-{K}_{3}^{2}-{\cos }^{2}{{\rm{\Phi }}}_{2}}\\ \,=\sin ({\omega }_{0}{\rm{\Delta }}{\tau }_{1,2}+{\varphi }_{1}-{\varphi }_{2})\cdot \sin ({\omega }_{0}{\rm{\Delta }}{\tau }_{2,3}+{\varphi }_{2}-{\varphi }_{3}),\\ \sin ({\mathop{\phi }\limits^{ \sim }}_{3}+{\omega }_{0}{\tau }_{3}-{\varphi }_{3})=\frac{{K}_{3}}{{\sin {\rm{\Phi }}}_{3}}.\end{array}$$

Suppose the third pulse propagates in the same direction as the second one. Then we have:

51 $${\tau }_{3}=\frac{{L}_{\mathrm{1,3}}-z}{c},$$

where *L*_1,3_ is the spatial separation between the first and the third pulses at *t* = 0. The time delay Δ*τ*_2,3_ is thus constant for every coordinate *z* and we choose it to be:

52 $${\omega }_{0}{\rm{\Delta }}{\tau }_{\mathrm{2,3}}+{\varphi }_{2}-{\varphi }_{3}=\pi +2\pi m$$

with some integer *m*.

Then Eq. (50) yields:

53 $$\begin{array}{ccc}{K}_{3} & = & -\sin ({\omega }_{0}{\rm{\Delta }}{\tau }_{1,2}+{\varphi }_{1}-{\varphi }_{2}),\\ {\cos {\rm{\Phi }}}_{3} & = & 0,\\ \sin ({\mathop{\phi }\limits^{ \sim }}_{3}+{\omega }_{0}{\tau }_{3}-{\varphi }_{3}) & = & -\sin ({\omega }_{0}{\rm{\Delta }}{\tau }_{1,2}+{\varphi }_{1}-{\varphi }_{2})\\ \Rightarrow {\mathop{\phi }\limits^{ \sim }}_{3} & = & \frac{3{\omega }_{0}z}{c}-\frac{{\omega }_{0}{L}_{1,2}}{c}-\frac{{\omega }_{0}{L}_{1,3}}{c}+{\varphi }_{3}-{\varphi }_{1}+{\varphi }_{2}=\frac{3{\omega }_{0}z}{c}+\delta {\mathop{\phi }\limits^{ \sim }}_{3}.\end{array}$$

So the third pulse erases the inversion grating and creates a traveling wave of the polarization with the wavenumber 3*k*_0_. Here we introduced for convenience the aggregate phase shift $\delta {\tilde{\phi }}_{i}$ for the pulse *i*. We thus obtain after the fourth pulse:

54 $$\begin{array}{ccc}\quad \,\quad \,\quad \,\quad \,\quad \,{K}_{4} & = & {\sin {\rm{\Phi }}}_{3}\,\sin ({\mathop{\phi }\limits^{ \sim }}_{3}+{\omega }_{0}{\tau }_{4}-{\varphi }_{4})=\,\sin ({\mathop{\phi }\limits^{ \sim }}_{3}+{\omega }_{0}{\tau }_{4}-{\varphi }_{4}),\\ \quad \,\quad \,\quad \quad \,{\cos {\rm{\Phi }}}_{4} & = & \sqrt{1-{K}_{4}^{2}}\,\sin (\frac{\pi }{2})=\,\cos ({\mathop{\phi }\limits^{ \sim }}_{3}+{\omega }_{0}{\tau }_{4}-{\varphi }_{4}),\\ \sin ({\mathop{\phi }\limits^{ \sim }}_{4}+{\omega }_{0}{\tau }_{4}-{\varphi }_{4}) & = & \frac{{K}_{4}}{{\sin {\rm{\Phi }}}_{4}}=1.\end{array}$$

We also assume the third and fourth pulses propagating in the opposite directions, what implies:

55 $${\tau }_{4}=\frac{{L}_{\mathrm{1,4}}+z}{c},$$

where *L*_1,4_ stands for the initial spatial separation between the first and fourth pulses. From Eq. (54) it follows:

56 $$\begin{array}{ccc}{\cos {\rm{\Phi }}}_{4} & = & \cos (\frac{4{\omega }_{0}z}{c}+\frac{{\omega }_{0}{L}_{1,4}}{c}+\delta {\mathop{\phi }\limits^{ \sim }}_{3}-{\phi }_{4})=\,\cos (\frac{4{\omega }_{0}z}{c}+\delta {\mathop{\phi }\limits^{ \sim }}_{4}),\\ \,\,\,\,\,{\mathop{\phi }\limits^{ \sim }}_{4} & = & \frac{\pi }{2}+{\varphi }_{4}-\frac{{\omega }_{0}}{c}({L}_{1,4}+z),\end{array}$$

that is, we get a population inversion grating and a standing wave of polarization with the spatial frequency 4*k*_0_.

In a similar way, assuming the fourth and fifth pulses propagating in the same direction, so that:

57 $${\tau }_{5}=\frac{{L}_{\mathrm{1,5}}+z}{c},$$

and the time delay Δ*τ*_4,5_being constant for every *z* which we choose here to be:

58 $${\omega }_{0}{\rm{\Delta }}{\tau }_{\mathrm{4,5}}+{\varphi }_{4}-{\varphi }_{5}=\pi +2\pi m,$$

with some integer *m*, we obtain for the fifth pulse:

59 $$\begin{array}{ccc}{K}_{5} & = & {\sin {\rm{\Phi }}}_{4}\cdot \sin ({\mathop{\phi }\limits^{ \sim }}_{4}+{\omega }_{0}{\tau }_{5}-{\phi }_{5})=\,\sin (\frac{4{\omega }_{0}z}{c}+\delta {\mathop{\phi }\limits^{ \sim }}_{4})\cdot \\  &  & \sin (\frac{\pi }{2}+{\omega }_{0}{\rm{\Delta }}{\tau }_{4,5}+{\varphi }_{4}-{\varphi }_{5})=-\sin (\frac{4{\omega }_{0}z}{c}+\delta {\mathop{\phi }\limits^{ \sim }}_{4}),\\ {\cos {\rm{\Phi }}}_{5} & = & \sqrt{1-{K}_{5}^{2}}\,\sin (\frac{\pi }{2}+\arcsin (\frac{\cos \,{{\rm{\Phi }}}_{4}}{\sqrt{1-{K}_{5}^{2}}}))\\  & = & \sqrt{1-{K}_{5}^{2}-{\cos }^{2}{{\rm{\Phi }}}_{4}}=0,\\ \sin ({\mathop{\phi }\limits^{ \sim }}_{5}+{\omega }_{0}{\tau }_{5}-{\varphi }_{5}) & = & -\sin (\frac{4{\omega }_{0}z}{c}+\delta {\mathop{\phi }\limits^{ \sim }}_{4})\\ \Rightarrow {\mathop{\phi }\limits^{ \sim }}_{5} & = & -\frac{5{\omega }_{0}z}{c}+\delta {\mathop{\phi }\limits^{ \sim }}_{4}+{\varphi }_{5}-\frac{{\omega }_{0}{L}_{1,5}}{c}=-\frac{5{\omega }_{0}z}{c}+\delta {\mathop{\phi }\limits^{ \sim }}_{5}.\end{array}$$

So the fifth pulse erases the population inversion grating and creates a traveling wave of the polarization with the wavenumber 5*k*_0_.

Let us now extend these results for an arbitrary number of exciting pulses. Suppose that after the pulse $(n-\mathrm{1)}$ we have the medium uniformly excited with $n(z,t)=0$ and the traveling wave of the polarization with the wavenumber $p{k}_{0}$:

60 $$\begin{array}{ccc}{\cos {\rm{\Phi }}}_{n-1} & = & 0,\\ {\mathop{\phi }\limits^{ \sim }}_{n-1} & = & \mp \frac{p{\omega }_{0}z}{c}+\delta {\mathop{\phi }\limits^{ \sim }}_{n-1},\end{array}$$

where *p* is some positive and odd integer.

We suppose the *n*-th pulse propagating in the direction opposite to the (*n* − 1)-th pulse, so that:

61 $${\tau }_{n}=\frac{{L}_{\mathrm{1,}n}\mp z}{c},$$

where the positive sign is taken for the pulses propagating in the same direction as the first one and the negative one for the pulses propagating in the opposite direction.

Considering this, we obtain for the *n*-th pulse:

$$\begin{array}{ccc}{K}_{n} & = & {\sin {\rm{\Phi }}}_{n-1}\,\sin ({\mathop{\phi }\limits^{ \sim }}_{n-1}+{\omega }_{0}{\tau }_{n}-{\varphi }_{n})=\,\sin ({\mathop{\phi }\limits^{ \sim }}_{n-1}+{\omega }_{0}{\tau }_{n}-{\varphi }_{n})\\  & = & \sin (\mp \frac{(p+1){\omega }_{0}z}{c}+\delta {\mathop{\phi }\limits^{ \sim }}_{n-1}+\frac{{\omega }_{0}{L}_{1,n}}{c}-{\varphi }_{n})\\  & = & \sin (\mp \frac{(p+1){\omega }_{0}z}{c}+\delta {\mathop{\phi }\limits^{ \sim }}_{n}),\\ {\cos {\rm{\Phi }}}_{n} & = & \sqrt{1-{K}_{n}^{2}}\,\sin (\frac{\pi }{2})=\,\cos (\mp \frac{(p+1){\omega }_{0}z}{c}+\delta {\mathop{\phi }\limits^{ \sim }}_{n}),\\ \sin ({\mathop{\phi }\limits^{ \sim }}_{n}+{\omega }_{0}{\tau }_{n}-{\varphi }_{n}) & = & \frac{{K}_{n}}{{\sin {\rm{\Phi }}}_{4}}=1\\ \Rightarrow {\mathop{\phi }\limits^{ \sim }}_{n} & = & \frac{\pi }{2}+{\varphi }_{n}-{\omega }_{0}\frac{{L}_{1,n}\mp z}{c}.\end{array}$$

That is, the *n*-th pulse produces a population inversion grating and a standing wave of polarization with the spatial frequency $(p+\mathrm{1)}{k}_{0}$.

The (*n* + 1)-th and *n*-th pulses are assumed to propagate in the same direction:

62 $${\tau }_{n+1}=\frac{{L}_{\mathrm{1,}n+1}\mp z}{c},$$

so that the time delay ${\rm{\Delta }}{\tau }_{n,n+1}$ is constant for every coordinate *z* and selected to be:

63 $${\omega }_{0}{\rm{\Delta }}{\tau }_{n,n+1}+{\varphi }_{n}-{\varphi }_{n+1}=\pi +2\pi m$$

with some integer *m*.

For the action of this (*n* + 1)-th pulse we then obtain:

64 $$\begin{array}{ccc}{K}_{n+1} & = & {\sin {\rm{\Phi }}}_{n}\,\sin (\mathop{\phi }\limits^{ \sim }+{\omega }_{0}{\tau }_{n+1}-{\varphi }_{n+1})\\  & = & \sin (\mp \frac{(p+1){\omega }_{0}z}{c}+\delta {\mathop{\phi }\limits^{ \sim }}_{n})\cdot \\ \sin (\frac{\pi }{2}+{\omega }_{0}{\rm{\Delta }}{\tau }_{n,n+1}+{\varphi }_{n}-{\varphi }_{n+1}) & = & -\sin (\mp \frac{(p+1){\omega }_{0}z}{c}+\delta {\mathop{\phi }\limits^{ \sim }}_{n}),\\ {\cos {\rm{\Phi }}}_{n+1} & = & \sqrt{1-{K}_{n+1}^{2}}\,\sin (\frac{\pi }{2}+\arcsin (\frac{{\cos {\rm{\Phi }}}_{n}}{\sqrt{1-{K}_{n+1}^{2}}}))\\  & = & \sqrt{1-{K}_{n+1}^{2}-{\cos }^{2}{{\rm{\Phi }}}_{n}}=0,\\ \sin ({\mathop{\phi }\limits^{ \sim }}_{n+1}+{\omega }_{0}{\tau }_{n+1}-{\varphi }_{n+1}) & = & -\sin (\mp \frac{(p+1){\omega }_{0}z}{c}+\delta {\mathop{\phi }\limits^{ \sim }}_{n})\\ \Rightarrow {\mathop{\phi }\limits^{ \sim }}_{n+1} & = & -\mp \frac{(p+2){\omega }_{0}z}{c}-\delta {\mathop{\phi }\limits^{ \sim }}_{n}+{\varphi }_{n+1}-\frac{{\omega }_{0}{L}_{1,n+1}}{c}\\  & = & \pm \frac{(p+2){\omega }_{0}z}{c}+\delta {\mathop{\phi }\limits^{ \sim }}_{n+1}.\end{array}$$

That is, the (*n* + 1)-th pulse erases the population inversion grating and induces a traveling wave of polarization with the wavenumber $(p+\mathrm{2)}{k}_{0}$. For illustration purposes the results of the analysis performed above are summarized in Table 1.

**Table 1:** Summary of polarization and population inversion gratings dynamics for the train of long pulses.

Pulse	Direction	Delay	Polarization, ${{\boldsymbol{\rho }}}_{{\bf{12}}}({\boldsymbol{z}}){{\boldsymbol{e}}}^{{\boldsymbol{-}}{\boldsymbol{i}}{\omega }_{{\boldsymbol{0}}}{\boldsymbol{t}}}$	Population inversion, *n*(*z*)
1	→	—	${e}^{-\frac{i{\omega }_{0}z}{c}+i{\varphi }_{1}}$	0
2	←	—	$\sin (-\frac{2{\omega }_{0}z}{c}+\delta {\mathop{\phi }\limits^{ \sim }}_{2}){e}^{\frac{i\pi }{2}+i{\varphi }_{2}+\frac{i{\omega }_{0}}{c}(z-{L}_{1,2})}$	$\cos (-\frac{2{\omega }_{0}z}{c}+\delta {\mathop{\phi }\limits^{ \sim }}_{2})$
3	←	${\omega }_{0}{\rm{\Delta }}{\tau }_{\mathrm{2,3}}+{\varphi }_{2}-{\varphi }_{3}=\pi +2\pi m$	${e}^{\frac{3i{\omega }_{0}z}{c}+i\delta {\tilde{\phi }}_{3}}$	0
4	→	—	$\sin (\frac{4{\omega }_{0}z}{c}+\delta {\mathop{\phi }\limits^{ \sim }}_{4}){e}^{\frac{i\pi }{2}+i{\varphi }_{4}-\frac{i{\omega }_{0}}{c}({L}_{1,4}+z)}$	$\cos (\frac{4{\omega }_{0}z}{c}+\delta {\mathop{\phi }\limits^{ \sim }}_{4})$
5	→	${\omega }_{0}{\rm{\Delta }}{\tau }_{\mathrm{4,5}}+{\varphi }_{4}-{\varphi }_{5}=\pi +2\pi m$	${e}^{-\frac{5i{\omega }_{0}z}{c}+i\delta {\tilde{\phi }}_{5}}$	0
…				
*n* − 1	→	—	${e}^{\mp \frac{pi{\omega }_{0}z}{c}+i\delta {\tilde{\phi }}_{n-1}}$	0
*n*	←	—	$\sin (\mp \frac{(p+1){\omega }_{0}z}{c}+\delta {\mathop{\phi }\limits^{ \sim }}_{n}){e}^{\frac{i\pi }{2}+i{\varphi }_{n}-\frac{i{\omega }_{0}}{c}({L}_{1,n}\mp z)}$	$\cos (\mp \frac{(p+1){\omega }_{0}z}{c}+\delta {\mathop{\phi }\limits^{ \sim }}_{n})$
*n* + 1	←	${\omega }_{0}{\rm{\Delta }}{\tau }_{n,n+1}+{\varphi }_{n}-{\varphi }_{n+1}=\pi +2\pi m$	${e}^{\pm \frac{(p+\mathrm{2)}i{\omega }_{0}z}{c}+i\delta {\tilde{\phi }}_{n+1}}$	0

Since in Eq. (64) we obtained the same expression as in Eq. (60) but with (*p* + 2) instead of *p*, we can continue the iteration procedure Eqs (60–64). It is also worth noting that the polarization waves Eqs (60), (64) induced by the *n* − 1-th and the (*n* + 1)-th pulse, respectively, propagate in opposite directions.

#### A train of ultrashort pulses

We turn now to the case Eqs (35–41) describing very short intense pulses. According to Eq. (40) we get:

65 $$\begin{array}{rcl}{B}_{n+1} & = & {\sin {\rm{\Phi }}}_{n}\,\cos ({\phi }_{n}+{\omega }_{0}{\rm{\Delta }}{\tau }_{n,n+1}),\\ {\cos {\rm{\Phi }}}_{n+1} & = & \sqrt{1-{B}_{n+1}^{2}}\,\sin (\pm 2{\rm{\Theta }}+\arcsin (\frac{{\cos {\rm{\Phi }}}_{n}}{\sqrt{1-{B}_{n+1}^{2}}})),\\ \cos \,{\phi }_{n+1} & = & \frac{{B}_{n+1}}{{\sin {\rm{\Phi }}}_{n+1}}\mathrm{.}\end{array}$$

We suppose as before that the resonant medium is initially uninverted, which means $A=\frac{1}{2},{{\rm{\Phi }}}_{0}=0$ and ${B}_{0}=0$. Let us assume that all the excitation pulses have the same pulse area equal now to ${{\rm{\Theta }}}_{i}=\frac{\pi }{4}$. We remind that the pulse area is defined in this case using Eq. (41).

Taking ${\phi }_{0}=0$ and the plus sign in Eq. (65), we obtain for the state of the medium modified by the first pulse:

66 $$\begin{array}{ccc}{B}_{1} & = & 0,\\ {\cos {\rm{\Phi }}}_{1} & = & \sin (\frac{\pi }{2}+\arcsin \,1)=\,\sin \,\pi =0,\\ \cos \,{\phi }_{1} & = & \frac{{B}_{1}}{{\sin {\rm{\Phi }}}_{1}}=0.\end{array}$$

That is, the first pulse fully saturates the medium.

Furthermore, considering now a spatially extended medium we assume (exactly as in the previous subsection):

67 $${\tau }_{1}=\frac{z}{c}\mathrm{.}$$

The resulting phase shift of the polarization is given as:

68 $$\phi (t)={\phi }_{1}+{\omega }_{0}(t-{\tau }_{1})=\frac{\pi }{2}+{\omega }_{0}(t-\frac{z}{c}),$$

corresponding to a traveling wave of polarization with the wavenumber:

$${k}_{0}=\frac{{\omega }_{0}}{c}\mathrm{.}$$

For the second pulse coming with time delay ${\rm{\Delta }}{\tau }_{\mathrm{1,2}}$ after the first one, we obtain:

$$\begin{array}{ccc}{B}_{2} & = & {\sin {\rm{\Phi }}}_{1}\,\cos ({\phi }_{1}+{\omega }_{0}{\rm{\Delta }}{\tau }_{1,2})=-\sin \,{\omega }_{0}{\rm{\Delta }}{\tau }_{1,2},\\ {\cos {\rm{\Phi }}}_{2} & = & \cos \,{\omega }_{0}{\rm{\Delta }}{\tau }_{1,2}\,\sin (\frac{\pi }{2}+\arcsin \,0)=\,\cos \,{\omega }_{0}{\rm{\Delta }}{\tau }_{1,2},\\ \cos \,{\phi }_{2} & = & \frac{{B}_{2}}{{\sin {\rm{\Phi }}}_{2}}=-1,\end{array}$$

so this pulse induces a periodic grating of population inversion as i﻿t﻿ is seen from the expression for *Δτ*_1,2_ given below by Eq. (﻿70).

Namely, we assume as before that the first and second pulses propagate in the opposite directions, so that:

69 $${\tau }_{2}=\frac{{L}_{\mathrm{1,2}}-z}{c},$$

where *L*_1,2_ is the spatial separation between the pulses at the moment when the first one starts to excite the medium. Then

70 $${\rm{\Delta }}{\tau }_{\mathrm{1,2}}=\frac{{L}_{\mathrm{1,2}}-2z}{c}\mathrm{.}$$

Given that, Eq. (69) means that the second pulse induces  a periodic grating of population inversion together with a standing wave of polarization with the phase distribution:

71 $$\phi (t)={\phi }_{2}+{\omega }_{0}(t-{\tau }_{2})=\pi +\frac{{\omega }_{0}}{c}(z-{L}_{\mathrm{1,2}})+{\omega }_{0}t\mathrm{.}$$

For the third pulse coming with time delay Δ*τ*_2,3_ after the second one, we obtain:

$$\begin{array}{ccc}{B}_{3} & = & {\sin {\rm{\Phi }}}_{2}\,\cos ({\phi }_{2}+{\omega }_{0}{\rm{\Delta }}{\tau }_{2,3})=-\sin \,{\omega }_{0}{\rm{\Delta }}{\tau }_{1,2}\,\cos \,{\omega }_{0}{\rm{\Delta }}{\tau }_{2,3},\\ {\cos {\rm{\Phi }}}_{3} & = & \sqrt{1-{B}_{3}^{2}}\,\sin (\frac{\pi }{2}+\arcsin (\frac{{\cos {\rm{\Phi }}}_{2}}{\sqrt{1-{B}_{3}^{2}}}))\\  & = & \sqrt{1-{B}_{3}^{2}-{\cos }^{2}{{\rm{\Phi }}}_{2}}=\,\sin \,{\omega }_{0}{\rm{\Delta }}{\tau }_{1,2}\,\sin \,{\omega }_{0}{\rm{\Delta }}{\tau }_{2,3},\\ \cos \,{\phi }_{3} & = & \frac{{B}_{3}}{{\sin {\rm{\Phi }}}_{3}}.\end{array}$$

Suppose, the second and  the third pulses propagate in the same direction. Then we have:

72 $${\tau }_{3}=\frac{{L}_{\mathrm{1,3}}-z}{c},$$

where *L*_1,3_ is the initial spatial separation between the first and the third pulses. The time delay ${\rm{\Delta }}{\tau }_{\mathrm{2,3}}$ is thus constant for every point $z$ and we select it as before to be an odd multiple of $\pi$:

73 $${\omega }_{0}{\rm{\Delta }}{\tau }_{\mathrm{2,3}}=\pi +2\pi m,$$

for an arbitrary integer *m*. Then Eq. (72) yields:

$$\begin{array}{ccc}{B}_{3} & = & \sin \,{\omega }_{0}{\tau }_{1,2},\\ {\cos {\rm{\Phi }}}_{3} & = & 0,\\ \cos \,{\phi }_{3} & = & \sin \,{\omega }_{0}{\tau }_{1,2}\\ \Rightarrow \phi (t) & = & {\phi }_{3}+{\omega }_{0}(t-{\tau }_{3})=\frac{\pi }{2}-{\omega }_{0}{\tau }_{1,2}-\frac{{\omega }_{0}}{c}({L}_{1,3}-z)+{\omega }_{0}t\\  & = & \frac{\pi }{2}+\frac{{\omega }_{0}}{c}(3z-{L}_{1,2}-{L}_{1,3})+{\omega }_{0}t={\omega }_{0}(t+\frac{3z}{c})+\delta {\phi }_{3}.\end{array}$$

That is, the third pulse erases the inversion grating and creates a traveling wave of polarization with the wavenumber 3*k*_0_.

For the fourth pulse we obtain:

74 $$\begin{array}{ccc}{B}_{4} & = & {\sin {\rm{\Phi }}}_{3}\,\cos ({\phi }_{3}+{\omega }_{0}{\rm{\Delta }}{\tau }_{3,4})=\,\cos ({\phi }_{3}+{\omega }_{0}{\rm{\Delta }}{\tau }_{3,4}),\\ {\cos {\rm{\Phi }}}_{4} & = & \sqrt{1-{B}_{4}^{2}}\,\sin (\frac{\pi }{2})=\,\sin ({\phi }_{3}+{\omega }_{0}{\rm{\Delta }}{\tau }_{3,4}),\\ \cos \,{\phi }_{4} & = & \frac{{B}_{4}}{{\sin {\rm{\Phi }}}_{4}}=1.\end{array}$$

We assume the third and fourth pulses propagate in the opposite directions, what implies:

75 $${\tau }_{4}=\frac{{L}_{\mathrm{1,4}}+z}{c},$$

where *L*_1,4_ is the initial spatial separation between first and fourth pulses. Then, from Eq. (74) it follows:

$$\begin{array}{ccc}{\cos {\rm{\Phi }}}_{4} & = & \sin (\frac{4{\omega }_{0}z}{c}+\delta {\phi }_{3}+\frac{{\omega }_{0}{L}_{1,4}}{c})=\,\sin (\frac{4{\omega }_{0}z}{c}+\delta {\phi }_{4}),\\ \phi (t) & = & {\phi }_{4}+{\omega }_{0}(t-{\tau }_{4})={\omega }_{0}(t-\frac{z+{L}_{1,4}}{c}).\end{array}$$

That is, we obtain now a population inversion grating and a standing wave of polarization with the spatial frequency 4*k*_0_.

In a similar way, assuming the fourth and fifth pulses propagating in the same direction, so that:

76 $${\tau }_{5}=\frac{{L}_{\mathrm{1,5}}+z}{c},$$

and thus the time delay ${\rm{\Delta }}{\tau }_{\mathrm{4,5}}$ is constant for every *z* which we choose to be:

77 $${\omega }_{0}{\rm{\Delta }}{\tau }_{4,5}=\pi +2\pi m$$

for an arbitrary integer *m*, we obtain for the state of the medium after the fifth pulse:

$$\begin{array}{ccc}{B}_{5} & = & {\sin {\rm{\Phi }}}_{4}\,\cos ({\phi }_{4}+{\omega }_{0}{\rm{\Delta }}{\tau }_{4,5})=\,\cos ({\phi }_{3}+{\omega }_{0}{\rm{\Delta }}{\tau }_{3,4})\cdot \\ \cos ({\phi }_{4}+{\omega }_{0}{\rm{\Delta }}{\tau }_{4,5}) & = & \cos (\frac{4{\omega }_{0}z}{c}+\delta {\phi }_{4})\cdot \cos ({\omega }_{0}{\rm{\Delta }}{\tau }_{4,5})=-\cos (\frac{4{\omega }_{0}z}{c}+\delta {\phi }_{4}),\\ {\cos {\rm{\Phi }}}_{5} & = & \sqrt{1-{B}_{5}^{2}}\,\sin (\frac{\pi }{2}+\arcsin (\frac{{\cos {\rm{\Phi }}}_{4}}{\sqrt{1-{B}_{5}^{2}}}))\\  & = & \sqrt{1-{B}_{5}^{2}-{\cos }^{2}{{\rm{\Phi }}}_{4}}=0,\\ \cos \,{\phi }_{5} & = & \frac{{B}_{5}}{{\sin {\rm{\Phi }}}_{5}}=-\cos (\frac{4{\omega }_{0}z}{c}+\delta {\phi }_{4})\\ \Rightarrow \phi (t) & = & {\phi }_{5}+{\omega }_{0}(t-{\tau }_{5})=\pi -\frac{4{\omega }_{0}z}{c}-\frac{{L}_{1,5}+z}{c}\\  &  & -\delta {\phi }_{4}+{\omega }_{0}t={\omega }_{0}(t-\frac{5z}{c})+\delta {\phi }_{5}.\end{array}$$

That is, the fifth pulse erases the population inversion grating and creates a traveling wave of polarization with the wavenumber $5{k}_{0}$.

Let us extend these results to an arbitrary number of the pulses. Suppose that after the $(n-\mathrm{1)}$-th pulse we have  the  medium uniformly saturated with a traveling wave of polarization with the wavenumber $p{k}_{0}$:

$$\begin{array}{ccc}{\rm{c}}{\rm{o}}{\rm{s}}\,{{\rm{\Phi }}}_{n-1} & = & 0,\\ {\phi }_{n-1} & = & \mp \frac{p{\omega }_{0}z}{c}+\pi -\delta {\phi }_{n-2},\\ \phi (t) & = & {\phi }_{n-1}+{\omega }_{0}(t-{\tau }_{n-1})=\mp \frac{p{\omega }_{0}z}{c}+\pi -\delta {\phi }_{n-2}\\ -\frac{{L}_{1,n-1}\pm z}{c}+{\omega }_{0}t & = & {\omega }_{0}(t\mp \frac{(p+1)z}{c})+\delta {\phi }_{n-1},\end{array}$$

where *p* is some positive even integer.

We now suppose that the *n*-th pulse propagates in the direction opposite to the $(n-\mathrm{1)}$-th pulse, so that:

78 $$\begin{array}{rcl}{\tau }_{n} & = & \frac{{L}_{\mathrm{1,}n}\mp z}{c},\\ {\rm{\Delta }}{\tau }_{n-\mathrm{1,}n} & = & \frac{{L}_{\mathrm{1,}n}-{L}_{\mathrm{1,}n-1}\mp 2z}{c},\end{array}$$

where the positive sign is taken for the pulses propagating in the same direction as the first one and the negative one for the pulses propagating in the opposite direction. With this assumption we obtain for the next (*n*-th) pulse:

$$\begin{array}{ccc}{B}_{n} & = & {\sin {\rm{\Phi }}}_{n-1}\,\cos ({\phi }_{n-1}+{\omega }_{0}{\rm{\Delta }}{\tau }_{n-1,n})=\,\cos ({\phi }_{n-1}+{\omega }_{0}{\rm{\Delta }}{\tau }_{n-1,n}),\\ {\cos {\rm{\Phi }}}_{n} & = & \sqrt{1-{B}_{n}^{2}}\,\sin (\frac{\pi }{2})=\,\sin ({\phi }_{n-1}+{\omega }_{0}{\rm{\Delta }}{\tau }_{n-1,n})\\  & = & \sin (\mp \frac{(p+2){\omega }_{0}z}{c}+\delta {\phi }_{n-1}+\frac{{\omega }_{0}({L}_{1,n}-{L}_{1,n-1})}{c})\\  & = & \sin (\mp \frac{(p+2){\omega }_{0}z}{c}+\delta {\varphi }_{n}),\\ \cos \,{\phi }_{n} & = & \frac{{B}_{n}}{{\sin {\rm{\Phi }}}_{n}}=1\\ \Rightarrow \phi (t) & = & {\phi }_{n}+{\omega }_{0}(t-{\tau }_{n})={\omega }_{0}(t-\frac{{L}_{1,n}\mp z}{c}).\end{array}$$

That is, the *n*-th pulse produces a population inversion grating and a standing wave of polarization with the spatial frequency $(p+\mathrm{2)}{k}_{0}$.

The next $(n+\mathrm{1)}$-th pulse propagates in the same direction as the *n*-th one:

79 $${\tau }_{n+1}=\frac{{L}_{\mathrm{1,}n+1}\mp z}{c},$$

so that the time delay ${\rm{\Delta }}{\tau }_{n,n+1}$ is constant for every *z* and we choose it to be:

80 $${\omega }_{0}{\rm{\Delta }}{\tau }_{n,n+1}=\pi +2\pi m,$$

where *m* is an arbitrary integer.

For the action of this $(n+\mathrm{1)}$-th pulse we then obtain:

81 $$\begin{array}{ccc}{B}_{n+1} & = & {\sin {\rm{\Phi }}}_{n}\,\cos ({\phi }_{n}+{\omega }_{0}{\rm{\Delta }}{\tau }_{n,n+1})\\  & = & -\cos (\mp \frac{(p+2){\omega }_{0}z}{c}+\delta {\phi }_{n}),\\ {\cos {\rm{\Phi }}}_{n+1} & = & \sqrt{1-{B}_{n+1}^{2}}\,\sin (\frac{\pi }{2}+\arcsin (\frac{{\rm{\cos }}\,{{\rm{\Phi }}}_{n}}{\sqrt{1-{B}_{n+1}^{2}}}))\\  & = & \sqrt{1-{B}_{n+1}^{2}-{{\rm{\cos }}}^{2}{{\rm{\Phi }}}_{n}}=0,\\ \cos \,{\phi }_{n+1} & = & \frac{{B}_{n+1}}{{\sin {\rm{\Phi }}}_{n+1}}=-\cos (\mp \frac{(p+2){\omega }_{0}z}{c}+\delta {\phi }_{n})\\ \Rightarrow \phi (t) & = & {\phi }_{n+1}+{\omega }_{0}(t-{\tau }_{n+1})=\pi -\mp \frac{(p+2){\omega }_{0}z}{c}\\  &  & -\delta {\phi }_{n}-\frac{{\omega }_{0}}{c}({L}_{1,n+1}\mp z)+{\omega }_{0}t={\omega }_{0}(t\pm \frac{(p+3)z}{c})+\delta {\phi }_{n+1},\end{array}$$

so $(n+\mathrm{1)}$-th pulse erases the population inversion grating and induces a traveling wave of polarization with the wavenumber $(p+\mathrm{3)}{k}_{0}$. The obtained results are summarized in Table 2.

**Table 2:** Summary of polarization and population inversion gratings dynamics for the train of ultrashort pulses.

Pulse	Direction	Delay	Polarization, ${{\boldsymbol{\rho }}}_{{\bf{12}}}({\boldsymbol{z}}){{\boldsymbol{e}}}^{{\boldsymbol{-}}{\boldsymbol{i}}{{\boldsymbol{\omega }}}_{{\bf{0}}}{\boldsymbol{t}}}$	Population inversion, *n*(*z*)
	→	—	${e}^{-\frac{i{\omega }_{0}z}{c}+\frac{i\pi }{2}}$	0
	←	—	$\sin (-\frac{2{\omega }_{0}z}{c}+\delta {\phi }_{2}){e}^{\frac{i{\omega }_{0}}{c}(z-{L}_{12})}$	$\cos (-\frac{2{\omega }_{0}z}{c}+\delta {\phi }_{2})$
	←	${\omega }_{0}{\rm{\Delta }}{\tau }_{\mathrm{2,3}}=\pi +2\pi m$	${e}^{\frac{3i{\omega }_{0}z}{c}+i\delta {\varphi }_{3}}$	0
	→	—	$\cos (\frac{4{\omega }_{0}z}{c}+\delta {\phi }_{4}){e}^{-\frac{i{\omega }_{0}}{c}({L}_{1,4}+z)}$	$\sin (\frac{4{\omega }_{0}z}{c}+\delta {\phi }_{4})$
	→	${\omega }_{0}{\rm{\Delta }}{\tau }_{\mathrm{4,5}}=\pi +2\pi m$	${e}^{-\frac{5i{\omega }_{0}z}{c}+i\delta {\phi }_{5}}$	0
…				
*n* − 1	→	—	${e}^{\mp \frac{(p+\mathrm{1)}i{\omega }_{0}z}{c}+i\delta {\varphi }_{n-1}}$	0
*n*	←	—	$\cos (\mp \frac{(p+2){\omega }_{0}z}{c}+\delta {\phi }_{n}){e}^{-\frac{i{\omega }_{0}}{c}({L}_{1,n}\mp z)}$	$\sin (\mp \frac{(p+2){\omega }_{0}z}{c}+\delta {\phi }_{n})$
*n* + 1	←	${\omega }_{0}\Delta {\tau }_{n,n+1}=\pi +2\pi m$	${e}^{\pm \frac{(p+\mathrm{3)}i{\omega }_{0}z}{c}+i\delta {\varphi }_{n+1}}$	0

Again, as for the case of the short pulses, we can continue the iteration Eqs (78–81) further with higher *n* and *p*. In general, although the analytics is quite different for the trains of the short and of the long pulses, the resulting behavior is very similar. As we see in the next subsection, the same behavior is found in the direct numerical simulations.

### Generation spatial gratings and their control

In the previous subsection we showed that the counter-propagating pulses sent at some delay change the state of the population inversion periodically with this delay time in every point of the medium, which can lead to a grating in a spatially extended medium. To provide numerical simulations, we extend our model with a wave equation describing propagation effects. That is, we consider the case of a spatially-extended medium and space- and time-dependent electric field $E(z,t)$ (we consider here one-dimensional case with a propagation coordinate *z*), which we describe by equations:

82 $$\frac{d{\rho }_{12}(z,t)}{dt}=-\frac{{\rho }_{12}(z,t)}{{T}_{2}}+i{\omega }_{0}{\rho }_{12}(z,t)-\frac{i{d}_{12}}{\hslash }n(z,t)E(z,t),$$

83 $$\frac{d}{dt}n(z,t)=-\frac{n(z,t)-{n}_{0}}{{T}_{1}}+\frac{4{d}_{12}E(z,t)}{\hslash }{\rm{Im}}({\rho }_{12}(z,t)),$$

84 $$\frac{{\partial }^{2}E(z,t)}{\partial {z}^{2}}-\frac{1}{{c}^{2}}\frac{{\partial }^{2}E(z,t)}{\partial {t}^{2}}=\frac{4\pi }{{c}^{2}}\frac{{\partial }^{2}P(z,t)}{\partial {t}^{2}}\mathrm{.}$$

The first pair of equations Eqs (82 and 83) are the extended versions of the Bloch equations Eqs (1 and 2) which take relaxation terms into account. The nondiagonal element of the density matrix ${\rho }_{12}(z,t)$, and the population inversion $n(z,t)={\rm{\Delta }}\rho (z,t)={\rho }_{11}(z,t)-{\rho }_{22}(z,t)$ now depend not only on time but also on the spatial coordinate. Furthermore, ${n}_{0}(z,t)$ is the equilibrium population difference and *T*_1_, *T*_2_ are the relaxation times. The same as Eqs (1 and 2), Eqs (82 and 83) describe the light matter interaction beyond RWA.

Finally, Eq. (84) is the nonlinear wave equation. The coupling to the medium is provided in right hand side, where $P(z,t)=2{N}_{0}{d}_{12}{\rm{Re}}{\rho }_{12}(z,t)$ is the medium polarization, with *N*_0_ being the concentration of the two-level atoms. By using the wave equation we avoid to use SVEA and thus allow description of few-cycle pulses propagating in arbitrary directions.

The wave equation was solved by the standard time domain finite-difference method (TDFD). The density matrix equations were solved by the 4-th order Runge-Kutta method. The full integration area in space was chosen to be 14*λ*_0_, and the resonant medium with the width 5*λ*_0_ was placed in the middle of the integration area. In the rest of the region vacuum was assumed. To create a sequence of pulses the reflective boundary conditions at the integration boundaries were imposed. That is, the pulses experience the full reflection at the boundaries of the integration area. This allowed us to send only two pulses into the system, the other pulses resulted from the reflection of the initial two. The pulses were excited in the system by imposing excitation in the form

85 $$E(t)={E}_{0}\exp (-(t-{\tau }_{i}){t}^{2}/{\tau }_{p}^{2})\sin ({\omega }_{0}(t-{\tau }_{i}))$$

on the boundaries, with the pulse duration ${\tau }_{p}$. In the subsequent figures, only the part of the whole area containing atoms will be shown.

**Figure 5:**
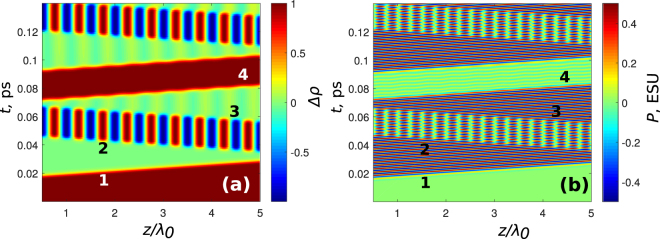
Spatial dependence of the population inversion ${\rm{\Delta }}\rho$ (**a**) and polarization *P* (**b**) in a resonant medium of $5{\lambda }_{0}$ length, demonstrating a spatial grating creation, erasing and deactivation. The input pulse amplitude ${E}_{0}=9\,\cdot \,{10}^{4}$ ESU, pulse duration ${\tau }_{p}=2.1\,\cdot \,{10}^{-15}$ s, ${d}_{12}=5\,\cdot \,{10}^{-18}$ ESU, ${N}_{0}={10}^{17}$ cm^−3^, ${T}_{1}={T}_{2}=1$ ns.

We now study the possibility of a grating creation and erasing by sending a pulse (1) with the area *π*/2 at ${\tau }_{1}=2.5{T}_{0}$ into the medium from the left; that is, an excitation in the form Eq. (85) was imposed at the left boundary. Furthermore, at the time ${\tau }_{2}=9.5{T}_{0}$ the pulse (2) with the area *π*/2 was sent from the right; that is, the excitation Eq. (85) was imposed on the right boundary. Both pulses, by the subsequent reflections from the integration boundaries, formed all the other pulses (2–4). The resulting dynamics is shown in Fig. 5. The pulse (1) transfers the atoms to the state with zero inversion (green region in Fig. 5a) and creates a traveling wave of polarization oscillating with frequency ${\omega }_{0}$ and wave vector ${k}_{0}=2\pi /{\lambda }_{0}$ (color line in Fig. 5b). Then, the second *π*/2 pulse (2), which has an amplitude opposite in sign to the pulse (1) (in order to compensate for the *π* phase shift upon the pulse reflection from the boundary) and propagates in the opposite direction, interacts with the wave of the polarization created by the first pulse and creates a grating of inversion with the wavevector 2*k*_0_ (period ${\lambda }_{0}\mathrm{/2}$), see Fig. 5a. Furthermore, the pulse (2) creates a standing wave of polarization, see Fig. 5b. This result is also in the qualitative agreement with the predictions of the previous section.

Next, the pulse (3) of area ${\rm{\Phi }}=\pi \mathrm{/2}$ propagating from right to left with the time delay ${\rm{\Delta }}{\tau }_{\mathrm{2,3}}=7{T}_{0}$ switches the medium back to zero inversion, thus the grating created by the pulse (2) is erased. Technically, the pulse (3) appears as a reflection of pulse (1) from the integration boundary. This agrees with our analytical predictions in the previous section. The pulse (3) creates a running wave of polarization with the wave vector *k*_0_ propagating with the velocity of light *c* from left to right, see Fig. 5b. Finally, the pulse (4) launched from left to right returns the media to the ground state (red region in Fig. 5a). The reversal action of the pulse is almost perfect, only a standing wave of polarization with very small amplitude is remaining. The deactivation process demonstrated here is also in agreement with the analytical results in the previous section. Further pulses shown in Fig. 5 just repeat the whole process of the grating creation and erasing from the very beginning.

**Figure 6:**
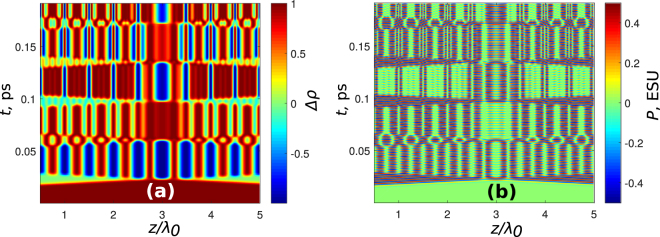
Breakdown of the grating management if the pulses cross in the nonlinear medium. Two pulses from the opposite directions with the parameters as in Fig. 5 cross at the point *z* = 3*λ*_0_. In (**a**) population difference and in (**b**) polarization is shown.

The process of grating creation and erasing demonstrated above depends critically on several important details. In particular, the pulses must not overlap in the medium, otherwise the grating is completely destroyed. The breakdown of the dynamics of the grating creation is shown in Fig. 6. Here, the counterpropagating pulses meet at the center of the integration area. After the overlap the dynamics of both population difference and polarization becomes rather complicated, with oscillations in space which are not anymore regular.

**Figure 7:**
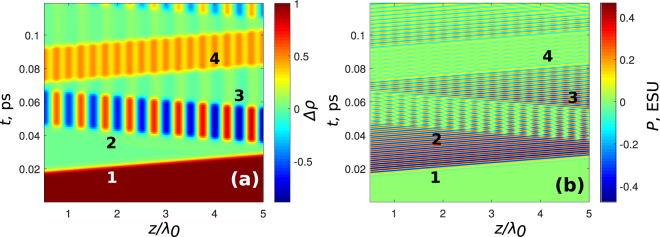
Disappearing of the grating if *T*_2_ is small. Here, the parameters of the system and of the pulses are the same as in Fig. 5 but *T*_2_ = 50 fs. In (**a**) population difference and in (**b**) polarization is shown.

Besides, it should be noted that the relaxation times play an important role in the process, because the grating creation here is based on the phase memory. If the phase decay times become comparable with the duration of the whole process of the grating formation, the grating disappears. This is illustrated in Fig. 7 for *T*_2_ = 50 fs; one can see that, although we are still able to create a grating because it happens on the times $t < {T}_{2}$, the erasure does not works properly anymore.

**Figure 8:**
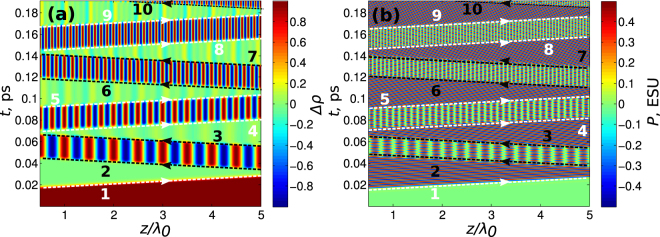
Multiplication of the grating period by modifying the pulse-to-pulse distance. Here, the parameters of the system and of the pulses are the same as in Fig. 5 but the pulse (3) (and thus all subsequent pulses with odd numbers) is send to the medium with the shift *T*_0_/2 compared to the corresponding pulse in Fig. 5. In (**a**) population difference and in (**b**) polarization is shown.

**Figure 9:**
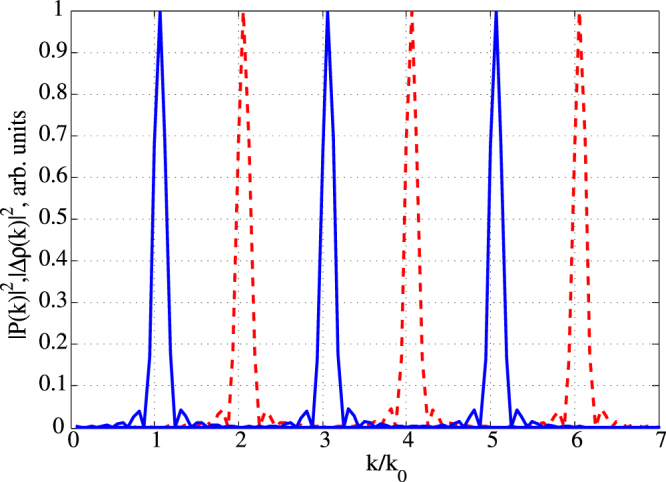
Spatial spectra of the polarization and population inversion gratings. Blue solid line: Spatial spectrum of the polarization gratings created after 1-th, 3-th and 5-th pulse in Fig. 8 (the peaks with increasing wavenumbers correspond to the increasing pulse number). Red dashed line: Spatial spectrum of the population inversion gratings created after 2-th, 4-th, and 6-th pulse in Fig. 8 (the peaks with increasing wavenumbers correspond to the increasing pulse number).

We can not only create and erase the gratings using the pulse sequences described above, but can also modify their period. In particular, if after the pulse (2), we send the pulse (3) with a delay shifted by *T*_0_/2 with respect to the previous case, the dynamics will be completely altered. Then, instead of erasing the grating, the pulse (4) will induce the population density grating of the period *λ*_0_/4. The subsequent pulses, if we repeat the procedure, will also not return the system to its initial state but will induce instead gratings with the period *λ*_0_/*n* for increasing *n*. Figure 8 illustrates the examples of the multiplication of the inversion (a) and polarization gratings (b) spatial frequency. In this example polarization gratings contains odd harmonics of wave vector *k*_0_: *k*_0_, 3*k*_0_, 5*k*_0_… The inversion gratings spatial frequencies contains the even harmonics of resonance wave vector: 2*k*_0_, 4*k*_0_, 6*k*_0…_ The spatial spectrum of the polarization and inversion gratings is plotted in Fig. 9 (see also Supplementary Material). An interesting point is that the polarization waveshape also contain harmonics proportional to *k*_0_. The details of this process are described elsewhere^40^.

**Figure 10:**
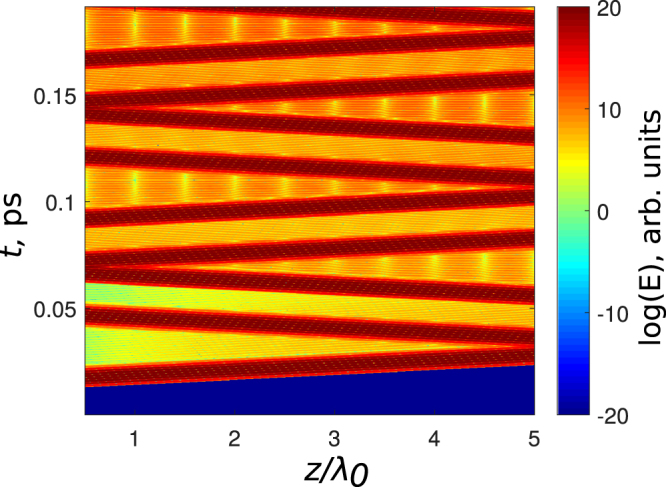
The field distribution $\mathrm{log}\,|E(z,t)|$ in the case of the dynamics shown in Fig. 8.

Finally, we remark that the inversion gratings shown here can radiate light because they contain excited atoms. An example of the radiating pattern for the case of Fig. 8 is shown in Fig. 10. One can see that between the pulses (the lines with highest intensity) there is also some radiation present. This radiation has rather limited intensity, because of the absence of the phase matching in this process.

### Beyond the two-level approximation

All the results above were obtained in the approximation of a two-level medium. The validity of this approximation is however becoming questionable as long as we consider very short pulses, so that the spectral pulse width includes several transitions. Thus, the approach here should be validated for ultrashort pulses taking into account the multilevel structure of the real atoms. Surprisingly, in the limit of single cycle pulse duration, the whole machinery related to the Rabi oscillations and related effects is rather stable with respect to the presence of other levels (and even whole bands in solid states), as it was shown both theoretically^33,55–57^ and experimentally^58^ in different situations.

**Figure 11:**
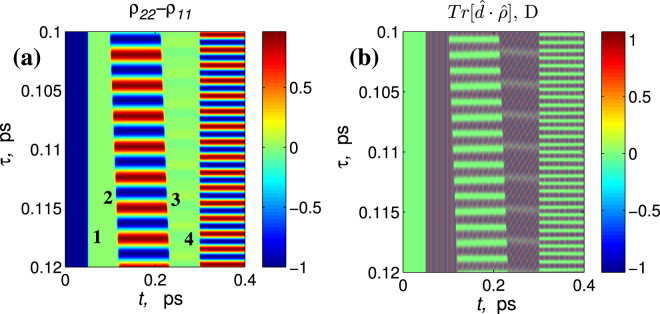
Dynamics of gratings of population inversion ${\rho }_{22}-{\rho }_{11}$ (**a**) and polarization per single atom $Tr[\hat{d}\cdot \hat{\rho }]$ (**b**) in a 4-level atom; pulse amplitude *E*_0_ = 2.26 $\cdot$ 10^5^ ESU, duration of pulses ${\tau }_{p}$ = 2.1 fs; delays between pulses: ${\rm{\Delta }}{\tau }_{\mathrm{2,3}}$ = 0.118 ps, ${\rm{\Delta }}{\tau }_{\mathrm{1,4}}$ = 0.25 ps.

In order to make sure that the dynamics of gratings will not disappear when considering realistic media with more complex energy structure we also performed exemplary calculations for a four-level medium. We chose the following wavelengths of the corresponding transitions in a four-level scheme: *λ*_12_ = 780.2 nm, *λ*_13_ = 420.2 nm, *λ*_24_ = 776.0 nm, *λ*_34_ = 5.2 *μ*m. Transition dipole moments were taken as follows: *d*_12_ = 2 D, *d*_13_ = 0.5 D, *d*_14_ = 0.05 D, *d*_23_ = 0.1 D, *d*_24_ = 0.5 D, *d*_34_ = 1 D, and the relaxation processes were assumed negligible over the considered time intervals. These values match in order-of-magnitude the ones typical for lowest excited states in alkali-metal atoms. Figure 11 shows the results of numerical simulations for the electric field of the form (2.1) with a carrier frequency of 800 nm.

The time evolution of the atomic wave function is described in this case by the following system of equations for the time-dependent coefficients ${a}_{n}(t)$ of the expansion into the series over eigenfunctions ${u}_{n}(\overline{r})$ of the unperturbed Hamiltonian^59^:

86 $$\begin{array}{rcl}\psi (t) & = & \sum _{n\mathrm{=1}}^{4}{a}_{n}(t){u}_{n}(\overline{r}){e}^{-\frac{i{E}_{n}t}{\hslash }},\\ {\dot{a}}_{n}(t) & = & \frac{i}{\hslash }\sum _{k\mathrm{=1}}^{4}{d}_{nk}{a}_{k}(t)E(t){e}^{i{\omega }_{kn}t},\\ {\omega }_{kn} & = & \frac{{E}_{k}-{E}_{n}}{\hslash },\end{array}$$

where *E*_*n*_ - energy of the *n*-th level, *d*_*nk*_ - transition dipole moment between levels *n* and *k*, ${\omega }_{kn}$ - frequency of the corresponding transition.

**Figure 12:**
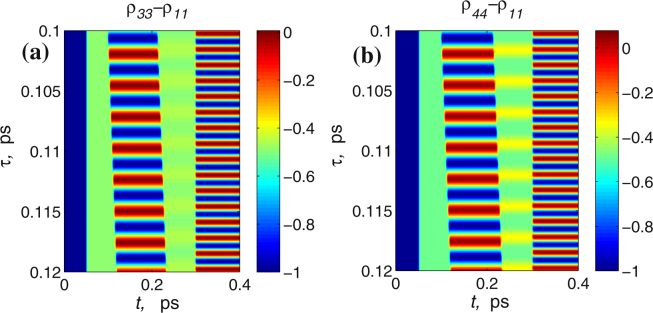
Dynamics of gratings of population inversion *ρ*_33_−*ρ*_11_ (**a**) and *ρ*_44_−*ρ*_11_ (**b**) in 4-level atom; all parameters are the same as in Fig. 11.

As can be seen from Fig. 11 the dynamics of gratings is similar to one obtained for a two-level medium in the previous sections. In particular, the pulse (2) creates a population inversion grating together with a traveling wave of polarization, the pulse (3) with the appropriate delay erases the grating while pulse (4) induces another grating with doubled spatial frequency. Similar dynamics, with only reduced amplitude, is observed for the population of other levels as shown in Fig. 12 desp﻿ite of their strong detuning from the﻿ resonance. It should be noted that the interaction of multiple levels can in general lead to multi-frequency modulation of the gratings and thus make the observed dynamics more complicated, but this question deserves special consideration which is outside the scope of this paper.

## Conclusions

In conclusion, we have studied the dynamics of the population gratings induced in a resonant medium by a train of non-overlapping optical pulses.

We discussed in detail the possibility of ultrafast creation, erasing and control of such gratings. Despite of the resonant character of the transitions in the medium, very short pulses up to a single cycle limit can be used for our scheme. Moreover, we have shown, using an example of a 4-level atom, that the method works surprisingly well beyond the two-level approximation even for single-cycle pulses. In contrast to the previous proposals the pulses do not need to be overlapped in the medium. Moreover, an﻿ overlap can even destroy the grating. The interaction between the pulses has an indirect character and is based on the phase memory, that is mediated by atomic polarization oscillations. We also were able to develop a theoretical approach describing such gratings, both in the case of long and short pulses.

The phenomenon considered here can be used for various applications in ultrafast optics, coherent control of the media properties, or attosecond science. In particular, population gratings can reflect light, which allows to use them in ultrafast optical devices such as deflectors^60–62^. Remarkably, the wave of polarization can be also considered as a moving Bragg mirrors, which can be used for the frequency conversion.

For experimental realization we could propose a few relevant candidates of resonant media where coherent pulse propagation was observed experimentally. Firstly, the atomic gases and vapours, such as Rb or Kr have explicit discrete energy levels and also can have relaxation times of the order of ns^18^. Then, semiconductor quantum dots can be thought as a suitable medium possessing a number of beneficial properties, like discrete atomic-type energy-level spectrum, large values of dipole moments (up to tens of Debays) and ultralong relaxation times at low temperatures^63–65^. In the mid-infrared and terahertz regions, intersubband transitions in semiconductor quantum heterostructures used as an active medium in quantum cascade lasers and having relatively long coherence times (values of *T*_2_ on the order of hundreds of fs) as well as very high dipole moments can be also considered. Specifically, Rabi oscillations were experimentally observed in quantum cascade lasers and the applicability of few-level model for the theoretical description of coherent interaction of ultrashort pulses with their active medium was demonstrated^66–68^.

## Electronic supplementary material

Supplementary material
